# Vinegar in the Treatment of Bowel Complaints

**Published:** 1885-01-20

**Authors:** 


### VINEGAR IN THE TREATMENT OF BOWEL COMPLAINTS.

In the October number of the Monthly, page 392, under the head of "A simple remedy for diarrhœa," the author says: "My simple remedy for diarrhœa, etc., is worth knowing and remembering." As the experience of the reader is invited, I will say that, for twenty-five years I have been in the habit of using vinegar, either alone or in combination with other remedies, with satisfactory results in bowel complaints of both children and adults; particularly where there is a tendency to muco-purulent or muco-sanguinous discharges. In dysentery I regard it as one of the very best remedies in combination with other therapeutic agents. I cannot now tell where I first got the idea, but it strikes me that I either read it in a medical journal or in some domestic work.

I have a very distinct recollection of a case treated in the year 1858 which left its lasting imprint on my mind. In the month of August of that year, I was called one evening to go twelve miles away to consult with two other physicians in a case of a child who was said to be dying of dysentery. On my arrival I found that the other physicians had abandoned the case as hopeless, and had gone away. The patient, a bright little girl of three summers, was, to all appearances, in a state of collapse. The extremities were cold, the mouth and fauces dry, the teeth covered with a reddish brown sordes, extreme thirst with, apparently, no choice of fluids, so it was drink ; the discharges from the bowels sanguino-purulent, and had been as frequent as every thirty minutes, during the latter part of that day ; no radial pulse ; heart's action quite feeble and irregular, skin pale and shrunken ; in fact, the signs of collapse were present. I ordered the child placed in blankets dipped in hot water, and given the following :

℞. Chlor. soda........................2 drachms.
Tr. capsici..............................2 fluid drachms.
Vinegar..................................2 fluid ounces.
Boiling water..........................3 fluid ounces. M.

Sig. : A teaspoonful every five minutes as hot as the patient could swallow it.

Before one-half of the mixture was given the child began to revive ; the extremities became warm, the skin became natural in appearance, the sordes was washed from the teeth by the vinegar, and she had no more action from the bowels for six hours, and then it was of a bilious character, mixed with the remnant of the offensive sanguino-purulent discharge. A few doses of quinine and dovers powder was all the medication she required in her after treatment, with proper directions as to diatetic rules. She made a speedy recovery.

I have treated many hundreds of cases of mucous diarrhœa and dysentery with vinegar variously combined, and am seldom disappointed in its action.

In low cases of dysenteric diseases I am rather partial to the above formula. I have given adults ounce-doses, repeated every few minutes. Frequently there is a thin, watery stool following the administration of this mixture, and when that is the case I follow it with a full opiate, which frequently ends the case.

I have not had as favorable results in the ordinary watery diarrhœa as in the dysenteric tendency of bowel complaints, but it usually puts an end to the griping and tormina.

In the dysentery of malarial origin, I have never found anything to stop the chilly sensations and the painful discharges so nicely as the mixture named above.

I can say truly " my simple remedy is worth knowing and remembering."—*Peoria Med. Monthly.*

